# Author Correction: Chromosome end protection by RAP1-mediated inhibition of DNA-PK

**DOI:** 10.1038/s41586-025-09121-9

**Published:** 2025-05-23

**Authors:** Patrik Eickhoff, Ceylan Sonmez, Charlotte E. L. Fisher, Oviya Inian, Theodoros I. Roumeliotis, Angela dello Stritto, Jörg Mansfeld, Jyoti S. Choudhary, Sebastian Guettler, Francisca Lottersberger, Max E. Douglas

**Affiliations:** 1 Telomere Biology Laboratory, The Institute of Cancer Research, London, UK; 2 Department of Biomedical and Clinical Sciences, Linköping University, Linköping, Sweden; 3 Structural Biology of Cell Signalling, The Institute of Cancer Research, London, UK; 4 Functional Proteomics, The Institute of Cancer Research, London, UK; 5 Post-translational Modifications and Cell Proliferation, The Institute of Cancer Research, London, UK

**Keywords:** Telomeres, Non-homologous-end joining

Correction to: *Nature* 10.1038/s41586-025-08896-1 Published online 16 April 2025

In the version of the article initially published, in the twelfth paragraph, the sentence “Binding of RAP1 to DNA may also restrict cNHEJ by preventing KU from translocating inwards, as proposed for budding yeast Rap1” and reference “Mattarocci, S. et al. Restriction on Ku’s inward translocation caps telomere ends. Preprint at *bioRxiv* 10.1101/2024.12.22.629684 (2024)” were missing and have now been added to the HTML and PDF versions of the article. In addition, Supplementary Table 1 was missing a biotin moiety from oligonucleotide PE3 and has been corrected.

