# Supplementary material for: Chromosome end protection by RAP1-mediated inhibition of DNA-PK
Source: Nature. 2025 Apr 16;642(8069):1090–6. doi: 10.1038/s41586-025-08896-1 (PMC12221994; doi:10.1038/s41586-025-08896-1)

nature portfolio

https://doi.org/10.1038/s41586-025-08896-1

Supplementary information

Chromosome end protection by RAP1-mediated inhibition of DNA-PK

In the format provided by the authors and unedited

Supplementary Table 1. Oligonucleotides used

PE1	CGGTACCCGGGGATCCTCTAGAGTCGACGAAGACTTAGGGTTAGGGTTAGGGT<br>TAGGGTTAGGGTTAGGGTTAGGGTTAGGGTTAGGGTTAGGGTTAGGGTTAGGG<br>TTAGGGTTAGGGTTAGGGTTAGGGTTAGGGTTAGGGTTAGGGTTAGGGTTAGG<br>GTTAGGGTTAGGGTTAGGGTTAGGGTTAGGGTTAGGGTTAGGGTTAGGGTTAG<br>GGTTAGGGTTAGGGTTAGGGTTAGGGTTAGGGTTAGGGTTAGGGTTAGGGTTA<br>GGGTTAGGGTTAGGGTTAGGGTTAGGGTTAGGGTTAGGGTTAGGGTTAGGGTT<br>AGGGTTAGGGTTAGGGTTAGGGTTAGGGTTAGGGTTAGGGTTAGGGTTAGGGT<br>TAGGGTTAGGGTTAGGG
PE2	CGGTACCCGGGGATCCTCTAGAGTCGACGAAGACCACCAGCTAGTACAACCAC<br>ATACTTTATGGAGAAATTTCAAAACGCAAAGAAGAACGAAGATAAACAAATTGCC<br>AAGTTTGAAAGCATGATGAATGCAAGAGTACATACGTTCAGTACCGATGAGAAG<br>AAATATGTGCCGATAATCACAAACGAATTAGAAAGCTTTTCAAATCTTTGGGTTAA<br>AAAGAGGTACATACCTGAAGATGACTTAAAACGGGCTTTGCATGAGATCAAAATC<br>CTTCGGTTGGGCAAACTTTTTGCTAAAATTCGCCCACCTAAATTTCAAGAGCCTG<br>AATACGCCAACTGGGCCACCGTAGGCCTCATTAGCCACAAATCGGACATCAAAT<br>TTAC
PE3	Biotin-CGTCTATATTCTATTGTCTCTTAGGGTTAGGGTTAGGGTTAGGGTTAGGGT<br>TAGGGTTAGGGTTAGGGTTAACATCAGTCTCACATAGATTAGCTCACGC
PE4	GCGTGAGCTAATCTATGTGAGACTGATGTTAACCCTAACCCTAACCCTAACCCTA<br>ACCCTAACCCTAACCCTAACCCTAAGAGACAATAGAATATAGACG
PE5	AGGGTTAGGGTTAGGGTTA

Supplementary Table 2. Constructs used

Insert in pACEBAC1 vector	UniProt ID	Tag	Mutation
KU70/80	P12956, XRCC6_HUMAN<br>P13010, XRCC5_HUMAN	FLAG, Ku70 N-terminal	WT
KU70/80 DE/KR	P12956, XRCC6_HUMAN<br>P13010, XRCC5_HUMAN	FLAG, Ku70 N-terminal	KU70 D327K + KU80 D496K, E499R point mutations
RAP1	Q9NYB0, TE2IP_HUMAN	Dual Strep, N-terminal	WT
RAP1 KR/DE	Q9NYB0, TE2IP_HUMAN	Dual Strep, N-terminal	K39D, R40E, R55E point mutations
RAP1 ΔBRCT	Q9NYB0, TE2IP_HUMAN	Dual Strep, N-terminal	M1-D108 deletion
RAP1 Δmyb	Q9NYB0, TE2IP_HUMAN	Dual Strep, N-terminal	Q128-D199 deletion
RAP1 ΔRCT	Q9NYB0, TE2IP_HUMAN	Dual Strep, N-terminal	S291-K399 deletion
RAP1 R133E	Q9NYB0, TE2IP_HUMAN	Dual Strep, N-terminal	R133E point mutation
Teb1:RAP1	Q10274, TEB1_SCHPO<br>Q9NYB0, TE2IP_HUMAN	Dual Strep, N-terminal	Teb1 22-227 fused to RAP1 1-399, with intervening sequence GGSGGLEVLFQ GPGGSGG

LIG4:RAP1 ΔBRCT	P49917, DNLI4_HUMAN<br>Q9NYB0, TE2IP_HUMAN	Dual Strep, N-terminal	Ligase IV 656-785 fused to RAP1 109-399
TRF2	Q15554, TERF2_HUMAN	Dual Strep, N-terminal	WT
TRF2 RBM	Q15554, TERF2_HUMAN	Dual Strep, N-terminal	L330R point mutation. (equivalent to L288R in a shorter TRF2 isoform)
TRF2 Δbasic	Q15554, TERF2_HUMAN	Dual Strep, N-terminal	M1-G86 deletion
TRF2 Δmyb	Q15554, TERF2_HUMAN	Dual Strep, N-terminal	T483-N542 deletion
TRF2 Δmyb Δbasic	Q15554, TERF2_HUMAN	Dual Strep, N-terminal	M1-G86, T483-N542 deletions
TRF2 Δbasic ΔTRFH	Q15554, TERF2_HUMAN	Dual Strep, N-terminal	M1-A315 deletion
shelterin:<br>TRF1<br>TRF2<br>RAP1<br>TPP1<br>POT1<br>TIN2	 P54274, TERF1_HUMAN<br>Q15554, TERF2_HUMAN<br>Q9NYB0, TE2IP_HUMAN<br>Q96AP0, ACD_HUMAN<br>Q9NUX5, POTE1_HUMAN<br>Q9BSI4, TINF2_HUMAN	 Strep, N-terminal on TINF2	
XRCC4/LIG4	Q13426, XRCC4_HUMAN<br>P49917, DNLIG4_HUMAN	Dual Strep, C-terminal on LIG4	

Supplementary Figure 1. Raw data and uncropped gels.

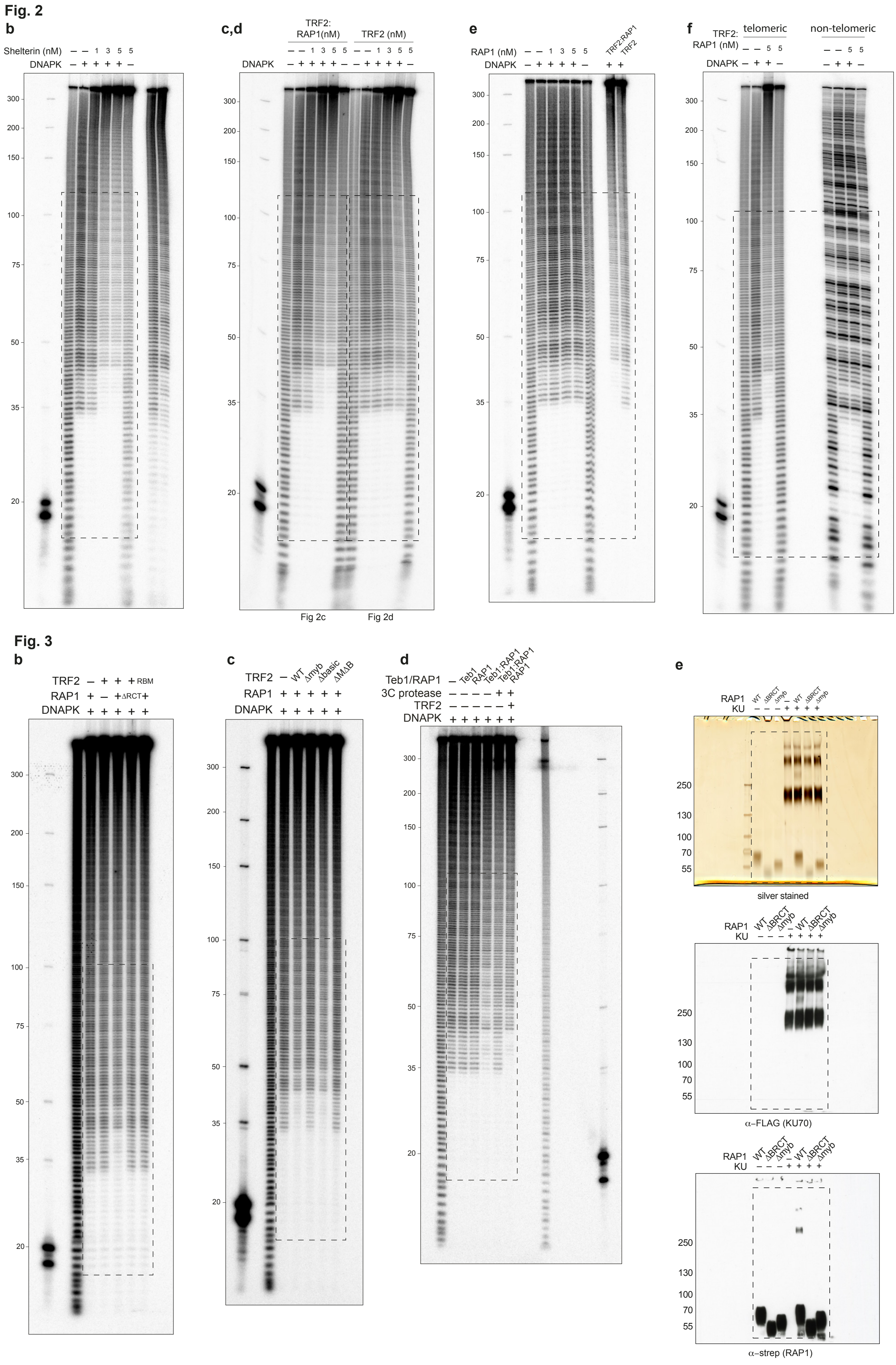

Fig. 3

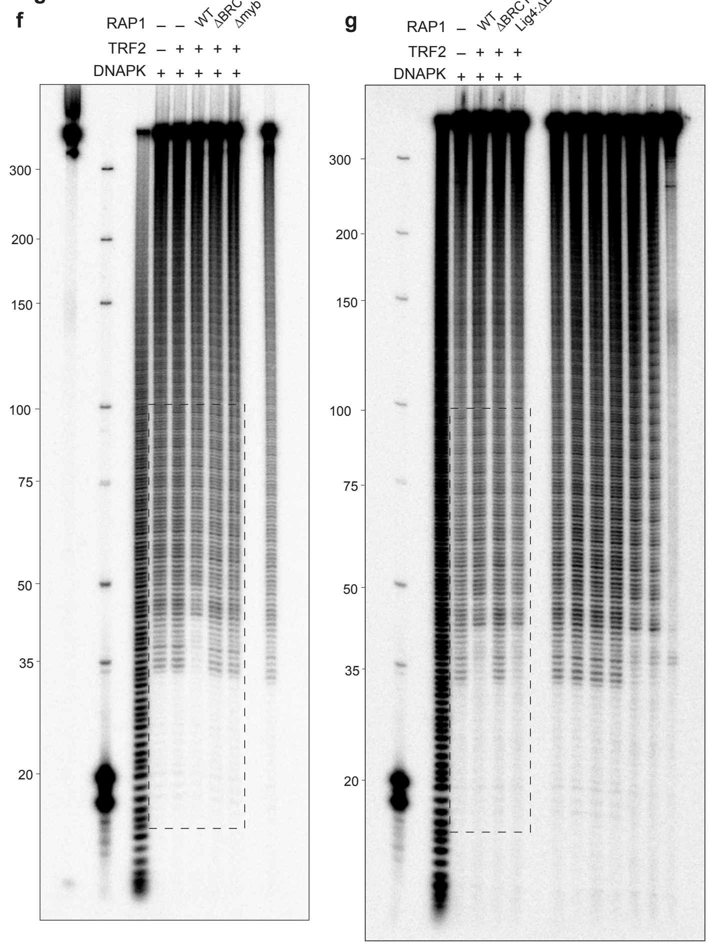

Fig. 4

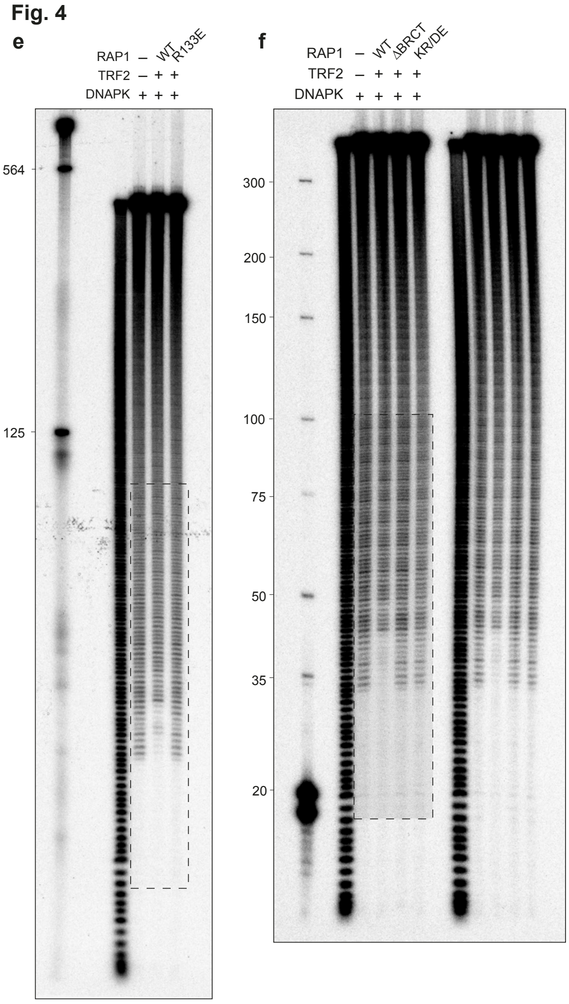

Fig. 4

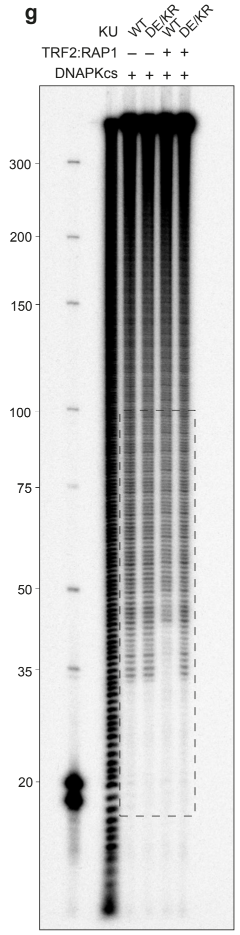

Fig 5

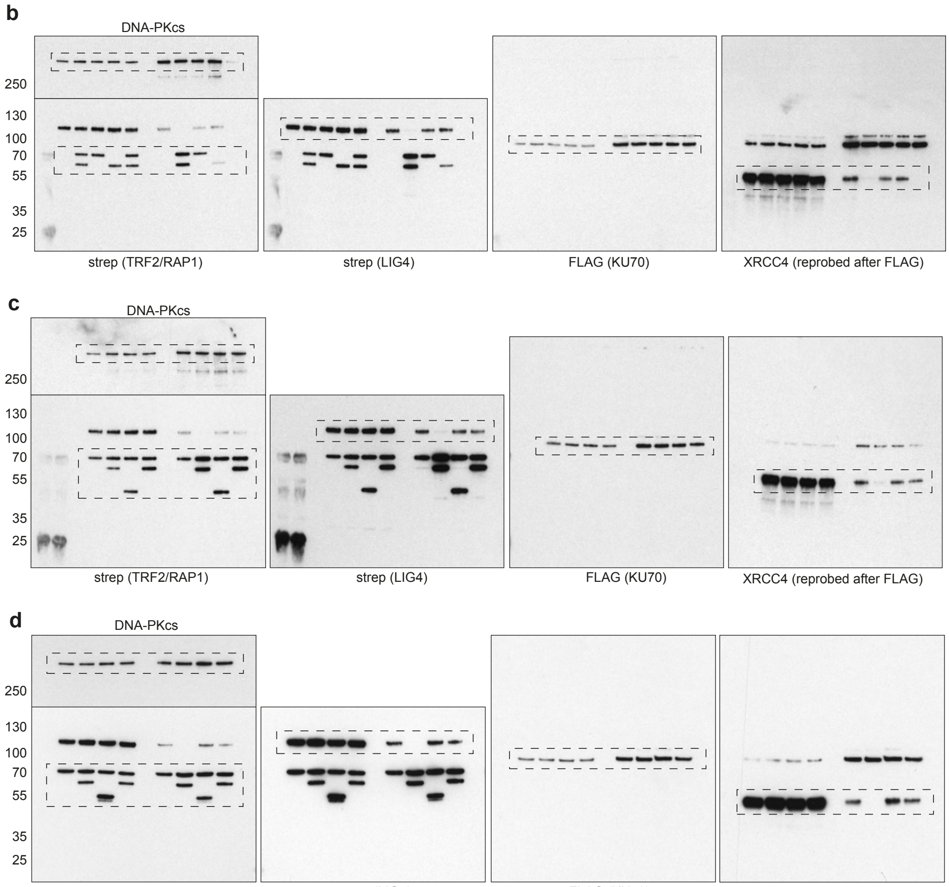

Extended Data Fig. 1

a

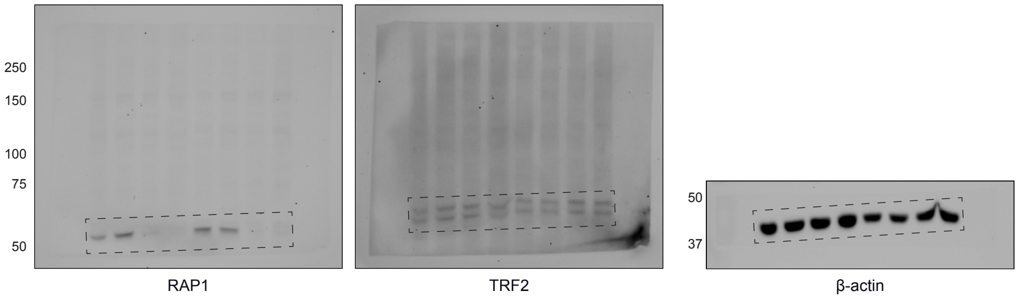

b

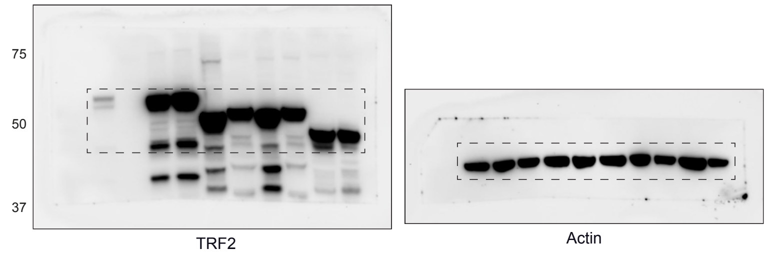

f

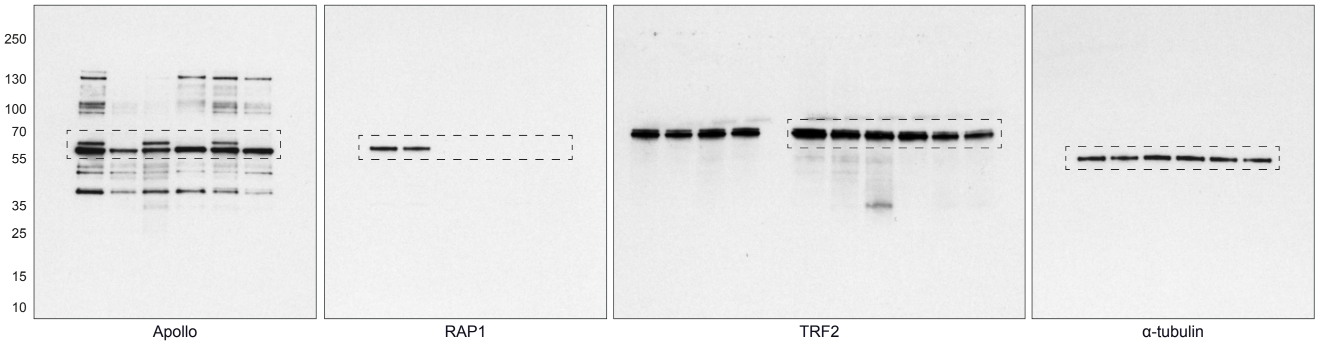

h

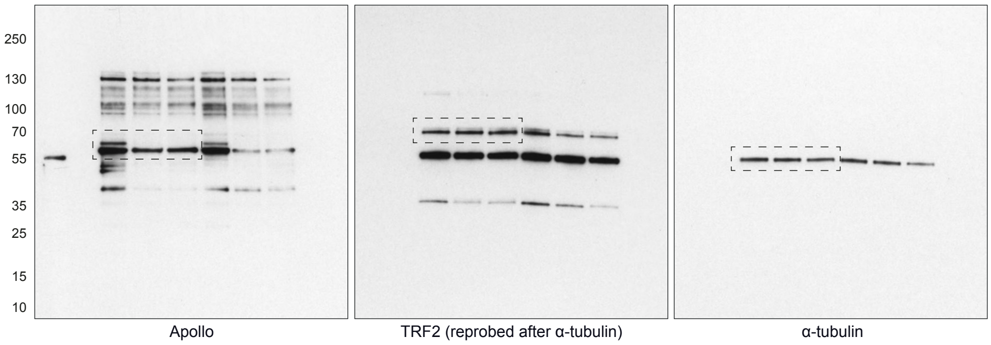

Extended Data Fig. 2

a,d

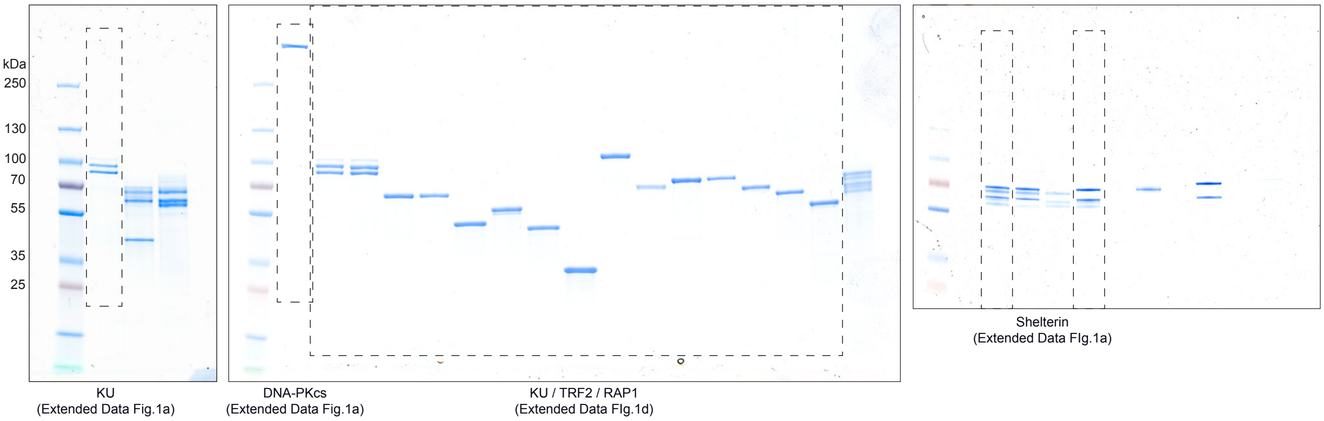

Extended Data Fig. 2

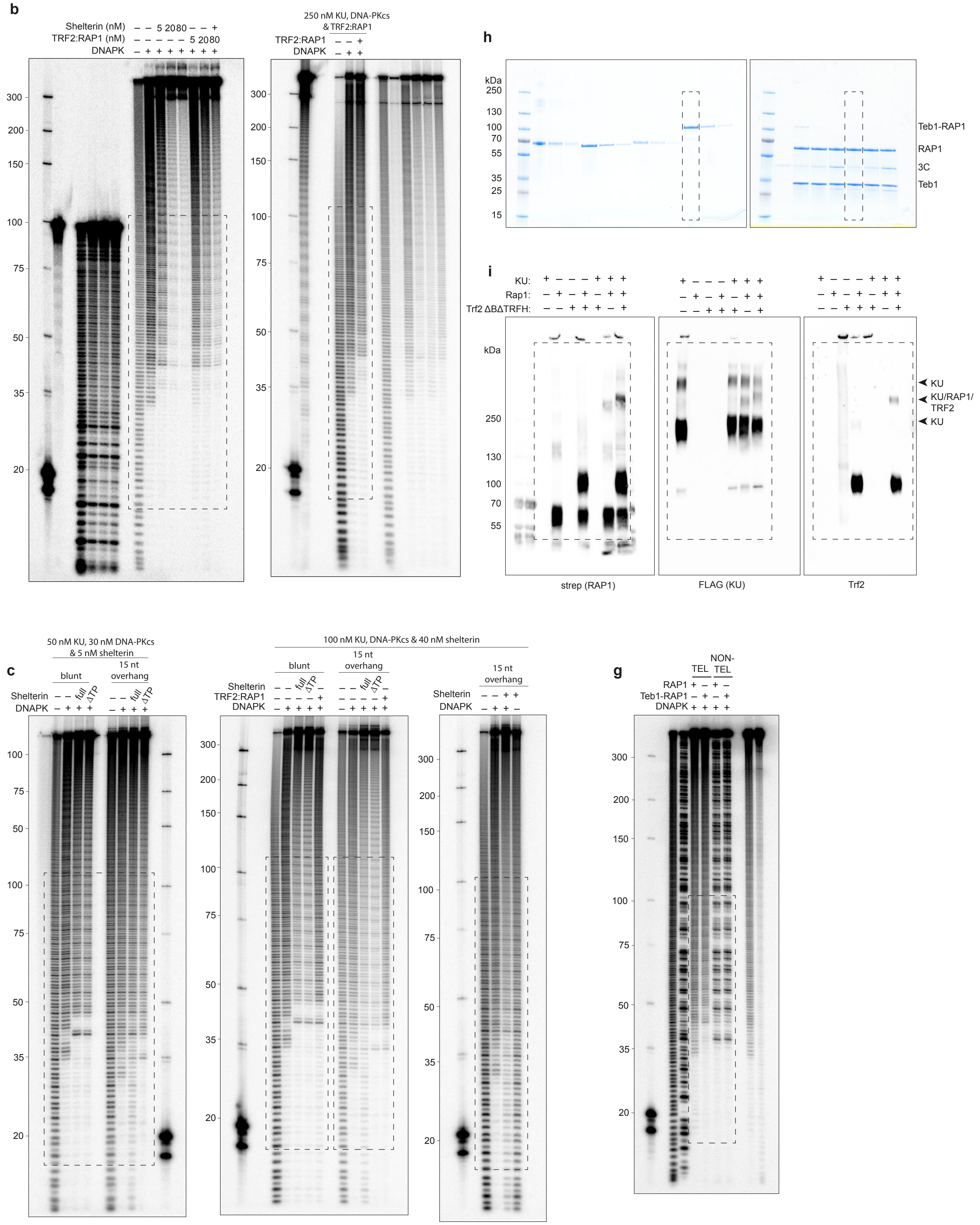

Extended Data Fig 6

c

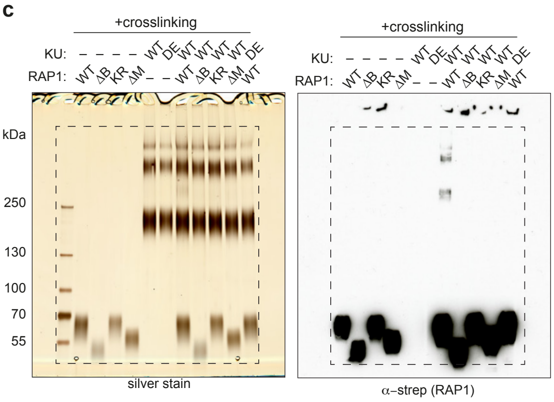

Extended Data Fig 7

a

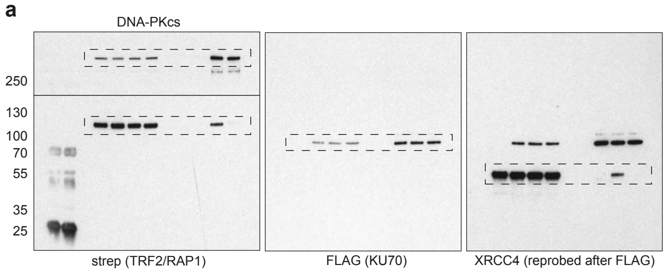

Extended Data Fig 7

b

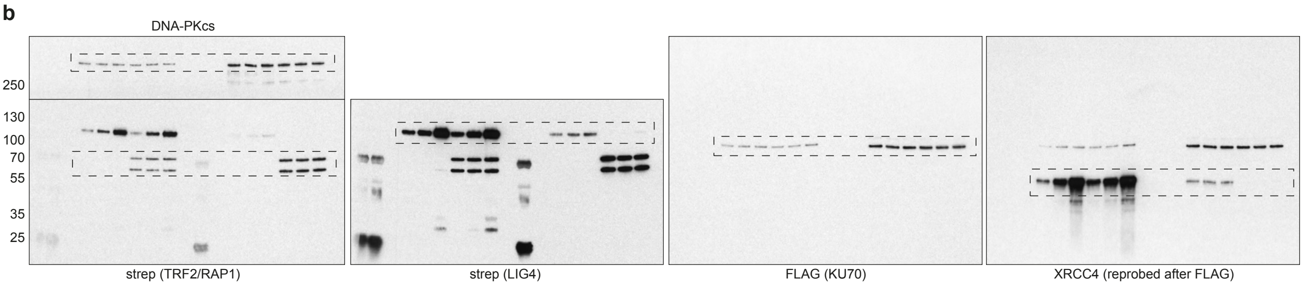

c

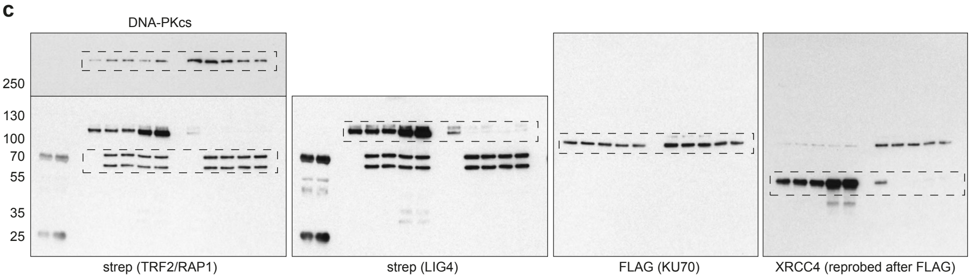

d

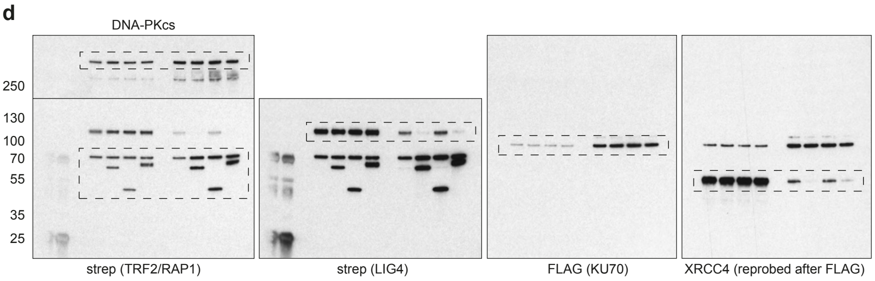

Extended Data Fig 8

a

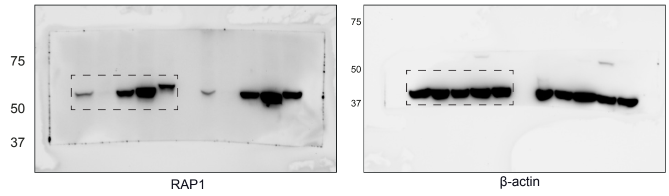

e

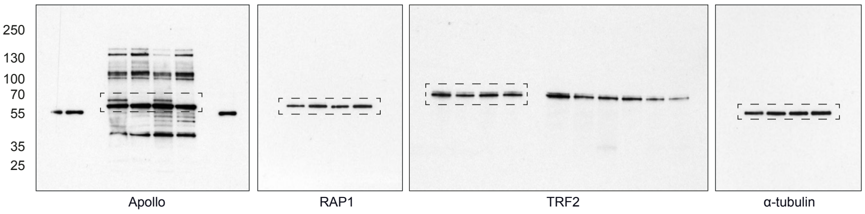

Supplement: Supplementary file 1 — This file contains Supplementary Tables 1 and 2 and Supplementary Fig. 1. Supplementary Table 1: Oligonucleotides used. Supplementary Table 2. Constructs used. Supplementary Fig. 1: Raw data and uncropped gels. [file 41586_2025_8896_MOESM1_ESM.pdf]
