# Supplementary material for: Chromosome end protection by RAP1-mediated inhibition of DNA-PK
Source: Nature. 2025 Apr 16;642(8069):1090–6. doi: 10.1038/s41586-025-08896-1 (PMC12221994; doi:10.1038/s41586-025-08896-1)
Supplement: Supplementary file 2 — Reporting Summary [file 41586_2025_8896_MOESM2_ESM.pdf]

nature portfolio

Corresponding author(s): Max E Douglas and Francisca Lottersberger

Last updated by author(s): Feb 25, 2025

Reporting Summary

Nature Portfolio wishes to improve the reproducibility of the work that we publish. This form provides structure for consistency and transparency in reporting. For further information on Nature Portfolio policies, see our Editorial Policies and the Editorial Policy Checklist.

Statistics

For all statistical analyses, confirm that the following items are present in the figure legend, table legend, main text, or Methods section.

n/a	Confirmed	
☐	☒	The exact sample size (*n*) for each experimental group/condition, given as a discrete number and unit of measurement
☐	☒	A statement on whether measurements were taken from distinct samples or whether the same sample was measured repeatedly
☐	☒	The statistical test(s) used AND whether they are one- or two-sided<br>*Only common tests should be described solely by name; describe more complex techniques in the Methods section.*
☒	☐	A description of all covariates tested
☒	☐	A description of any assumptions or corrections, such as tests of normality and adjustment for multiple comparisons
☐	☒	A full description of the statistical parameters including central tendency (e.g. means) or other basic estimates (e.g. regression coefficient) AND variation (e.g. standard deviation) or associated estimates of uncertainty (e.g. confidence intervals)
☐	☒	For null hypothesis testing, the test statistic (e.g. F, t, r) with confidence intervals, effect sizes, degrees of freedom and P value noted<br>*Give P values as exact values whenever suitable.*
☒	☐	For Bayesian analysis, information on the choice of priors and Markov chain Monte Carlo settings
☒	☐	For hierarchical and complex designs, identification of the appropriate level for tests and full reporting of outcomes
☒	☐	Estimates of effect sizes (e.g. Cohen's *d*, Pearson's *r*), indicating how they were calculated

Our web collection on statistics for biologists contains articles on many of the points above.

Software and code

Policy information about availability of computer code

Data collection	Cryo-EM data collection: ThermoFisher EPU v3.2<br>Phosphor imaging: Amersham TYPHOON Scanner Control Software v3.0.0.2<br>Nano differential scanning fluorimetry data collection: Tycho NT.6 v1.3.2.880<br>Microscopy: Slide book (3i), DeltaVision RT microscope system (GE Healthcare)<br>Gel and membrane scanning: ChemiDoc (Bio-Rad)
Data analysis	Structural data analysis: cryoSPARC v4.3.1, Phenix v1.20.1, Coot v0.9.8.92, Namdinator, UCSF ChimeraX v1.6.1, Alphafold 3<br>Nano differential scanning fluorimetry data analysis: Tycho NT.6 v1.3.2.880<br>Protein sequence alignment analysis: ProViz (online tool)<br>Image processing: ImageJ2 (FIJI) v2.14.0, FIJI v1.0, Adobe Photoshop v25.0.0, Adobe Illustrator v25.0.0<br>Metaphase spread graphs: GraphPad Prism (v9 or 10.0.0)

For manuscripts utilizing custom algorithms or software that are central to the research but not yet described in published literature, software must be made available to editors and reviewers. We strongly encourage code deposition in a community repository (e.g. GitHub). See the Nature Portfolio guidelines for submitting code & software for further information.

Data

Policy information about availability of data

All manuscripts must include a data availability statement. This statement should provide the following information, where applicable:

- Accession codes, unique identifiers, or web links for publicly available datasets
- A description of any restrictions on data availability
- For clinical datasets or third party data, please ensure that the statement adheres to our policy

The cryo-EM composite map of the RAP1:DNA-PK complex was deposited to the Electron Microscopy Data Bank under accession code EMD-19065. Corresponding atomic coordinates were deposited to the Protein Data Bank under PDB ID 8RD4. Constituent cryo-EM maps for locally refined KU:RAP1 and DNA-PKcs regions as well as a consensus map of the full complex were deposited under accession codes EMD-19249, EMD-19252 and EMD-19245 respectively. Mass spectrometry proteomics data have been deposited to the ProteomeXchange Consortium via the PRIDE59 partner repository with the dataset identifier PXD047643.

Research involving human participants, their data, or biological material

Policy information about studies with human participants or human data. See also policy information about sex, gender (identity/presentation), and sexual orientation and race, ethnicity and racism.

Reporting on sex and gender	N/A
Reporting on race, ethnicity, or other socially relevant groupings	N/A
Population characteristics	N/A
Recruitment	N/A
Ethics oversight	N/A

Note that full information on the approval of the study protocol must also be provided in the manuscript.

Field-specific reporting

Please select the one below that is the best fit for your research. If you are not sure, read the appropriate sections before making your selection.

☒ Life sciences ☐ Behavioural & social sciences ☐ Ecological, evolutionary & environmental sciences

For a reference copy of the document with all sections, see nature.com/documents/nr-reporting-summary-flat.pdf

Life sciences study design

All studies must disclose on these points even when the disclosure is negative.

Sample size	No statistical method was used to predetermine sample size. For chromosome fusion experiments, sample size was determined based on previous similar experiments: n=30-40 metaphases over 3-4 independent experiments (Lottersberger et al., Cell, 2015). For cryo-EM data collection, sample size was determined to obtain sufficient resolution for model building.
Data exclusions	For chromosome fusion experiments, metaphases where it was not possible to perform robust scoring (i.e. less than 10 chromosomes, too many overlapping chromosomes) were excluded. For cryo-EM image processing, particles that did not align into discernable 2D or 3D classes were excluded. No other data were excluded from experiments in the study.
Replication	All analyses were independently replicated a minimum of 3 times except experiments in Figure 3c as well as Extended Data Fig 1h,i; 2f,g and 7c which were replicated twice. All attempts at replication were successful.
Randomization	For cryo-EM volumes, final particle subsets were randomly split into two independent halves to calculate Fourier Shell Correlation. For other experiments, no randomization was applied. Appropriate controls were included where applicable.
Blinding	Mouse cell metaphase spreads were not scored blind as sample identity was obvious to the experienced investigator. Human metaphase spreads were scored blind. Blinding was not compatible with other experiments, which involved electrophoresis.

Reporting for specific materials, systems and methods

We require information from authors about some types of materials, experimental systems and methods used in many studies. Here, indicate whether each material, system or method listed is relevant to your study. If you are not sure if a list item applies to your research, read the appropriate section before selecting a response.

Materials & experimental systems

n/a	Involved in the study
☐	☒ Antibodies
☐	☒ Eukaryotic cell lines
☒	☐ Palaeontology and archaeology
☒	☐ Animals and other organisms
☒	☐ Clinical data
☒	☐ Dual use research of concern
☒	☐ Plants

Methods

n/a	Involved in the study
☒	☐ ChIP-seq
☒	☐ Flow cytometry
☒	☐ MRI-based neuroimaging

Antibodies

Antibodies used	Mouse monoclonal anti-DNA-PKcs, Invitrogen MA5-13238, clone 18-2, 1:100 dilution<br>Rabbit-polyclonal anti-RAP1, Bethyl A300-306A, 1:4000 dilution<br>Rabbit monoclonal anti-RAP1, Cell Signalling 5433, clone D9H4, 1:1000 dilution<br>Rabbit monoclonal anti-TRF2, Cell Signalling 13136, clone D1Y5D, 1:1000 dilution<br>Rabbit polyclonal anti-DCLRE1B, Atlas HPA064934, 1:100 dilution<br>Mouse monoclonal anti-XRCC4, Santa Cruz sc-271087, clone C-4, 1:500 dilution<br>Mouse monoclonal anti-β-actin, Cell Signalling 3700, clone 8H10D10, 1:4000 dilution<br>Mouse monoclonal anti-α-tubulin, Sigma T9026, clone DM1A, 1:1000 dilution<br>Mouse monoclonal anti-FLAG, Sigma F1804, clone M2, 1:1000 dilution<br>Rabbit polyclonal anti-Strep-tag II, Abcam ab76949, 1:1000 dilution<br>Goat polyclonal anti-Rabbit IgG HRP, Cell Signalling 7074<br>Horse anti-Mouse IgG HRP, Cell Signalling 7076<br>Donkey polyclonal anti-Rabbit IgG HRP, Cytiva NA934V<br>Goat polyclonal anti-Rabbit IgG HRP, Invitrogen 31460<br>Goat polyclonal anti-Mouse IgG HRP, Invitrogen 31430
Validation	Antibodies for mouse RAP1 (Cell Signalling 5433), mouse TRF2 (Cell Signalling 13136), human Apollo (Atlas HPA064934) and human RAP1 (Bethyl A300-306A) were validated in this study by Crispr-mediated knockout of target genes followed by western blotting, confirming loss of the relevant band. Other primary antibodies have been previously validated as follows:<br><br>anti-DNA-PKcs, Invitrogen MA5-13238 - validated by a strong band of the correct molecular weight in human LS174T cells<br>anti-XRCC4, Santa Cruz sc-271087 - validated by Western blotting against purified recombinant XRCC4 protein<br>anti-β-actin, Cell Signalling 3700 - validated by Western blotting against recombinant β-actin protein<br>anti-α-tubulin, Sigma T9026 - validated by a strong band of the correct molecular weight in mouse cell lysates and a characteristic tubulin immunofluorescence pattern in fixed human cells.<br>Anti-FLAG and anti-strep tag antibodies - validated by detection of purified recombinant FLAG or strep-tagged proteins

Eukaryotic cell lines

Policy information about cell lines and Sex and Gender in Research

Cell line source(s)	SV40-LT ApolloF/F Lig4+/+, ApolloF/F Lig4-/-, and Trf2F/FRosa26Cre-ERT1 MEFs were from Wu et al, Molecular Cell, 2010 and Lottersberger et al, Cell, 2015. 293T/17 [HEK 293T/17] (CRL-11268) and Phoenix ECO cells (CRL-3214) were from ATCC, Rockville, MD. RPE-1 cells were from Hegerat et al, EMBO J, 2020.
Authentication	RPE-1 cells were validated by whole genome sequencing. MEFs used in the study were automatically genotyped after isolation by TransnetYX for the presence of Apollo or TRF2 "Flox" alleles and/or Ligase IV deletion or RsCre. No cell line validation was performed for 293T or Phoenix ECO cells after acquisition.
Mycoplasma contamination	All cell lines in this study routinely tested negative for mycoplasma contamination.
Commonly misidentified lines (See ICLAC register)	No cell lines on the current ICLAC register of misidentified cell lines (version 13) were used in this study.

Plants

Seed stocks

Report on the source of all seed stocks or other plant material used. If applicable, state the seed stock centre and catalogue number. If plant specimens were collected from the field, describe the collection location, date and sampling procedures.

Novel plant genotypes

Describe the methods by which all novel plant genotypes were produced. This includes those generated by transgenic approaches, gene editing, chemical/radiation-based mutagenesis and hybridization. For transgenic lines, describe the transformation method, the number of independent lines analyzed and the generation upon which experiments were performed. For gene-edited lines, describe the editor used, the endogenous sequence targeted for editing, the targeting guide RNA sequence (if applicable) and how the editor was applied.

Authentication

Describe any authentication procedures for each seed stock used or novel genotype generated. Describe any experiments used to assess the effect of a mutation and, where applicable, how potential secondary effects (e.g. second site T-DNA insertions, mosiacism, off-target gene editing) were examined.
